# Comparison of the fracture resistance of the teeth prepared with ProTaper Universal, ProTaper Next, and ProTaper Gold rotary files

**DOI:** 10.1002/cre2.660

**Published:** 2022-09-02

**Authors:** Amin Salem Milani, Shabnam Ganjpour, Fatemeh Dehghani, Saeed Rahimi, Pouya Sabanik

**Affiliations:** ^1^ Endodontic Department, Faculty of Dentistry Tabriz University of Medical Sciences Tabriz Iran; ^2^ Private Practice Tabriz Iran; ^3^ Dental and Periodontal Research Centre Tabriz University of Medical Sciences Tabriz Iran; ^4^ Dental and Periodontal Research Center Tabriz University of Medical Sciences Tabriz Iran

**Keywords:** fracture resistance, ProTaper Gold (PTG), ProTaper Next (PTN), ProTaper Universal (PTU)

## Abstract

**Objectives:**

Root canal preparation can lead to cracks on the roots by creating stresses on the root canal walls, which decreases the fracture resistance of the tooth. The present study compared the fracture resistance of the teeth prepared by the ProTaper Universal (PTU), ProTaper Next (PTN), and ProTaper Gold (PTG) rotary file systems.

**Materials and Methods:**

Fifty‐six single‐canal premolar teeth were sectioned 14 mm from the root apex. The roots were standardized based on the buccolingual and mesiodistal diameter and randomly assigned to three experimental (*n* = 14) and one control group (*n* = 14). The teeth in three experimental groups were instrumented with PTU, PTN, and PTG rotary files. The roots in the control group were not instrumented. A vertical force was applied to each root in a universal testing machine until the root fractured. The data were statistically analyzed by one‐way analysis of variance.

**Results:**

There was no significant difference in the fracture resistance of the teeth between the control, PTU, PTN, and PTG groups (*p* = .115).

**Conclusions:**

Root canal preparation with ProTaper files manufactured with conventional NiTi (PTU) and heat‐treated alloys (PTN and PTG) did not affect the fracture resistance of teeth.

## 1 INTRODUCTION

Vertical root fracture (VRF) is an undesired event that strongly affects teeth prognosis. VRF is not an instant phenomenon and mainly results from the propagation of fine cracks and defects occurring during tooth function over time or due to various dental procedures (Shemesh et al., 2008; Soros et al., 2008). Endodontic treatments are among the main susceptibility factors for root cracks (Von Arx et al., 2021). Root canal preparation and obturation procedures might cause or increase cracks on root canal walls by creating stresses (Adorno et al., 2010; Bier et al., 2009).

Some studies have shown an increased prevalence of cracks in teeth prepared with NiTi rotary files (Nagendrababu & Ahmed, 2019) by exerting stress on root canal walls (Ingle et al., 2008).

The rotary files are mainly produced from NiTi alloys. In recent years, modifications have been made in NiTi alloys to increase the flexibility and decrease the fracture of rotary files; these modifications have focused on the conversion temperatures of different phases of the alloy (Peters et al., 2020). One of these changes is the heat treatment before or after manufacturing rotary files in different ways or temperatures that change the conversion temperatures of the austenite phase to the martensite phase and vice versa. The conventional NiTi alloy is in the austenite phase at room temperature, and the conversion temperature to the martensite phase is lower than the body temperature (Shen et al., 2011; Zhou et al., 2012). Superelasticity (SE) also known as pseudoelasticity is related to the thermoelastic martensitic transformation. SE of near‐equiatomic NiTi alloy arises from the reversible stress‐induced martensitic (SIM) transformation and depends on the temperature difference between the working temperature and the austenite finish temperature *A*_f_ (Zhou et al., 2012). The SE of NiTi rotary instruments provides improved access to curved root canals during the instrumentation, leading to their success in clinical practice.

M‐wires were introduced by heat treatment in 2007. Their conversion temperature to the martensite phase is higher than the room temperature. However, they are in a hybrid state at room temperature with mainly an austenite phase. The proportions of martensite are too low to develop superflexibility. Another rotary system introduced by heat treatment is CM‐wire files that undergo gold or blue heat treatment. In these files, the phase conversion temperature is higher than the body temperature, and unlike the files mentioned above, they are predominantly in the martensite phase at room temperature, with no superelasticity and spring‐back at room temperature. They have high flexibility and controlled memory (Plotino et al., 2014).

ProTaper Universal (PTU) rotary files (Dentsply‐Maillefer, Switzerland) have been made of conventional NiTi alloy. With the introduction of M‐wires, the Dentsply‐Maillefer Company introduced ProTaper Next (PTN) files made of M‐wire. After the introduction of the gold and blue treatments, the Dentsply‐Maillefer Company introduced its newer file, ProTaper Gold (PTG) in which the heat treatment is carried out after the grinding process (AlShwaimi, 2018; Özyürek et al., 2017).

Capar et al. (2014) showed that the dentinal cracks due to root canal preparation with PTN files were significantly less than those with PTU files, which was also confirmed by other studies (Miguéns‐Vila et al., 2017; Priya et al., 2014). However, in two separate studies, Karatas et al. showed that the number of dentin cracks created by PTU files was more than those produced by PTN and PTG files only in the apical third of the root, with no significant differences in the coronal or middle thirds (Karataş et al., 2015, 2016).

Since no study has evaluated the effects of these three different generations of rotary systems on the fracture resistance of teeth, the present study aimed to evaluate the fracture resistance of roots prepared with these different rotary systems.

## 2 MATERIALS AND METHODS

A total of 56 human premolar teeth extracted for orthodontic or periodontal reasons were selected for the study, after receiving informed consent from subjects, complying with the following inclusion criteria: single‐rooted teeth with a single canal and fully formed apices, without calcification and previous endodontic treatment as confirmed radiographically. The teeth had no coronal or radicular restorations or caries, or root curvature. The root length from the anatomic apex to CEJ was >14 mm.

The teeth were cleaned with a scaler under running water and stored in 0.1% thymol solution until use. All teeth were sectioned at 14 mm from the anatomic apex using a diamond‐coated bur under water cooling. Then the buccolingual (BL) and mesiodistal (MD) sizes of the teeth were determined in the coronal area of the sectioned root using a digital caliper. All roots were examined with a stereomicroscope (Nikon, Japan) under ×10 magnification to detect pre‐existing craze lines or cracks. A different set of samples was substituted for the roots with cracks or craze lines.

To simulate PDL (periodontal ligament), the root surfaces were covered with a layer of wax with a homogeneous thickness and mounted vertically within an acrylic resin block. Using hot water, the wax was removed, and the space left by the wax was filled with silicone impression material (Speedex, Coltene, Switzerland). BL and MD radiographs were prepared to ensure the vertical positioning of teeth.

### 2.1 Root canal preparation

The roots were randomly assigned to the following four groups:

**Group 1 (PTU)**: Instrumentation with PTU (Dentsply‐Maillefer, Switzerland) (*n* = 14): After determining the working length (WL) with a #15 K‐file and taking a control radiograph, SX, S1, S2, F1, F2, and F3 PTU files were used, according to the manufacturer's instructions. At the end of the preparation procedure, the F3 file reached the entire WL.

**Group 2 (PTN)**: Instrumentation with PTN (Dentsply‐Maillefer, Switzerland) rotary files (*n* = 14): After determining the WL and taking a control radiograph, X1, X2, and X3 files were used, respectively, according to the manufacturer's instructions. The X1 file was placed in the root canal up to half of the WL, and the remaining files were used for the WL.

**Group 3 (PTG)**: Instrumentation with PTG (Dentsply‐Maillefer, Switzerland) rotary files (*n* = 14): The root canal preparation procedure was similar to Group 1 (PTU).

**Group 4 (control)**: No root canal preparation was carried out.

In all the groups, after each file, the root canal was irrigated with 2 ml 1% sodium hypochlorite solution (NaOCl) (Clorox Corp., Oakland, CA, USA) that was used in a 2‐ml syringe with a 27‐G needle. To minimize operator variation, all the preparation steps were carried out by one operator.

### 2.2 Fracture resistance test

**Figure 1:**
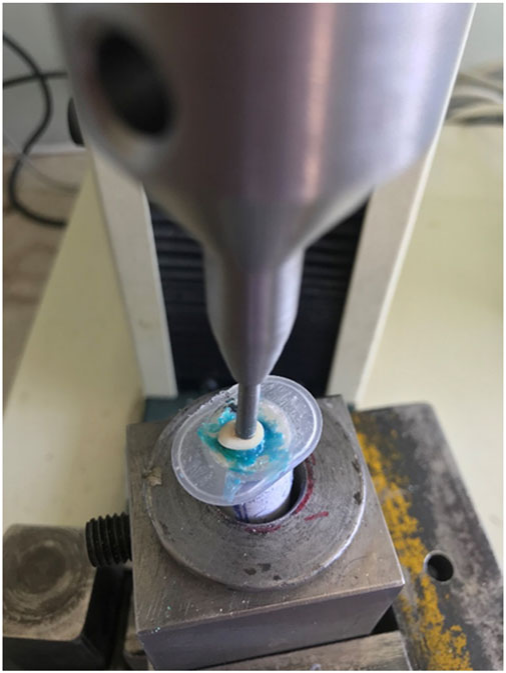
Fracture testing with a universal testing machine

The prepared roots were tested with a universal testing machine (Hounsfield, England) (Figure 1). A vertical force was applied to the root canal orifices with the round tip of the unit arm, 3 mm in diameter, with a 1 mm/min crosshead speed. The minimum force necessary for fracture was recorded in newtons (N).

### 2.3 Statistical analysis

The data were statistically analyzed using SPSS 20 software package (IBM Corp, Armonk, NY, USA). Kolmogorov−Smirnov test was used to evaluate the normality of data. One‐way analysis of variance was used to compare fracture resistance between study groups.

## 3 RESULTS

There was no significant difference in the BL and MD width of the roots between the different groups before preparation (Table 1).

**Table 1:** Comparison of the BL and MD widths between the study groups

Group	BL width	MD width
PTU	7.14	4.93
PTN	7.14	4.86
PTG	7.14	4.86
Control	7.07	4.93
p Value	.804	.755

*Note*: *p* Value: one‐way analysis of variance.

Abbreviations: BL, buccolingual; MD, mesiodistal; PTG, ProTaper Gold; PTN, ProTaper Next; PTU, ProTaper Universal.

Kolmogorov−Smirnov test showed normal distribution of data (*p* = .200). The means of fracture resistance values in the PTU, PTN, PTG, and control groups were 872.3, 1081.41, 1125.63, and 1085.82 N, respectively (Table 2). The comparison of the fracture resistance values did not reveal any significant differences between the study groups (*p* > .05).

**Table 2:** Comparison of fracture resistance (newton) between the study groups

Group	*n*	Mean	SD	*p* Value
PTU	14	872.3	203.46	
PTN	14	1081.41	322.22	.115
PTG	14	1125.63	245.26	
Control	14	1085.82	384.68	

*Note*: p Value: one‐way ANOVA.

Abbreviations: PTG, ProTaper Gold; PTN, ProTaper Next; PTU, ProTaper Universal.

## 4 DISCUSSION

The present study evaluated and compared the fracture resistance of teeth prepared with the PTU, PTN, and PTG rotary systems. These rotary systems are the different generations of rotary files manufactured by the Dentsply Company. PTU has been manufactured using the conventional NiTi alloy. PTN has been manufactured from M‐wire, which is a heat treatment NiTi alloy. In addition to the difference in the alloy, PTN also has a special off‐centered rectangular design (Capar et al., 2014). PTG and PTU are similar in design; however, PTG has been made from CM‐wire. Rotary systems manufactured using heat‐treated NiTi alloys, such as M‐wire or CM‐wire, have high flexibility. Therefore, they are expected to exert less pressure on tooth structures during root canal preparation (Karataş et al., 2016).

In the present study, the PDL stimulation was carried out to resemble a clinical situation (Çiçek et al., 2015; Karataş et al., 2015). In addition, the prepared root canals were not obturated so that the net effect of root canal preparation with rotary files could be compared with each other and with the control group. This way, the possible effect of the root canal obturation process on creating dentin cracks and fracture resistance was eliminated as a confounding factor.

The present study showed no significant differences in the fracture resistance of teeth prepared with different rotary systems. In other words, preparation with different ProTaper systems manufactured using different alloys did not decrease tooth resistance to fracture. Çiçek et al. (2015) compared the fracture resistance of teeth prepared with the PTN and PTU systems and reported no significant differences in their fracture resistance, consistent with the present study. In that study, the root canals were obturated with the single cone technique after preparation, and the fracture resistance of root‐filled teeth was higher than the control teeth that were prepared but not obturated.

Most of the studies evaluating the effect of rotary files on the fracture resistance of teeth have evaluated the occurrence of dentin cracks due to root canal preparation (Çiçek et al., 2015; Karataş et al., 2015; Miguéns‐Vila et al., 2017). These studies have evaluated the effects of different rotary files on the fracture resistance of teeth indirectly. Abou El Nasr and Abd El Kader (2014) claimed that evaluating dentinal cracks might be a good indicator of teeth fracture resistance. Some studies have compared the effect of root canal preparation with ProTaper rotary files with different alloys on creating dentinal cracks. Karatas et al. (2015) showed that the number of dentinal cracks due to root canal preparation with PTU files was significantly higher than this with the PTN system, and both systems created more cracks than the control group. Subsequent studies confirmed these findings (Capar et al., 2014; Karataş et al., 2015). In addition, Harandi and Ajinka in two separate studies showed that the PTN system resulted in more dentinal cracks than a control unprepared group (Harandi et al., 2017; Pawar et al., 2018). We used a fracture strength test to compare the fracture strength of teeth prepared by a different system. This method is more direct and clinically relevant compared to counting dentinal cracks.

Since the PTU and PTN systems also have different designs, the difference in creating dentinal cracks cannot be attributed only to the difference in their alloys, and the cumulative effect of both variables might have a role in such differences (Capar et al., 2014). Based on the results of previous studies, it might be concluded that the dentinal cracks created by the rotary systems are more than the unprepared control teeth. In addition, the ProTaper rotary systems with heat‐treated alloys, such as PTN and PTG, create fewer dentinal cracks in the apical area than the PTU system (Karataş et al., 2015, 2016).

The present study showed no significant differences between the study groups concerning fracture resistance. It is not possible to directly compare the results of the aforementioned studies with the present study due to differences in study methods. So more dentinal cracks found in the PTU system compared to the heat‐treated alloys did not necessarily decrease fracture strength in this group. This can be explained in different ways. First, in the present study, the fracture resistance in the PTU group was less than in the two other groups and the control group.

However, the difference was not significant, which might be attributed to the small sample size in the present study. Therefore, a study with a larger sample size is recommended. Second, the fracture resistance test was carried out immediately after root canal preparation in the present study. However, under clinical conditions, the tooth fracture process is not a sudden occurrence; instead, it occurs due to the propagation of fine cracks over time (Shemesh et al., 2008; Soros et al., 2008). Since it was not possible to carry out mechanical aging in the present study, it is recommended that after preparing the teeth, an aging process be carried out before the fracture strength test to better simulate the clinical conditions.

Third, as explained previously, the main difference in dentin crack formation between the different ProTaper systems is in the apical area (Karataş et al., 2015, 2016) while in the fracture resistance test, occlusal force is applied to fracture the teeth. Further studies are necessary to evaluate whether dentinal cracks in the apical area necessarily decrease the fracture resistance under occlusal forces or not. Furthermore, the effect of the different NiTi alloys would be more obvious on roots with curvatures; it is recommended to carry out future studies on teeth with curved roots.

ProTaper Ultimate is a further development of the ProTaper family, keeping the same philosophy and a similar technique compared to the previous generations of ProTaper, with substantial additional benefits like more flexibility and greater resistance to cyclic fatigue. So it is recommended to compare the fracture resistance of the teeth prepared with ProTaper Ultimate with the other ProTaper files in future studies. Meanwhile, the results of this study should be interpreted considering the limitation of in vitro studies.

## 5 CONCLUSIONS

Within the limitations of this study, it can be concluded that root canal preparation with ProTaper files manufactured with conventional NiTi (PTU) and heat‐treated alloys (PTN and PTG) did not affect the fracture resistance of teeth without root curvature.

## AUTHOR CONTRIBUTIONS

All authors participated in the conception, design of the study and proposal writing, and data collection. Amin Salem Milani and Fatemeh Dehghani analyzed the data and drafted the manuscript. Amin Salem Milani, Fatemeh Dehghani, Shabnam Ganjpour, Saeed Rahimi, and Pouya Sabanik reviewed the drafted manuscript. Amin Salem Milani and Fatemeh Dehghani revised the final manuscript. All authors read and approved the manuscript for submission.

## CONFLICT OF INTEREST

The authors declare no conflict of interest.

## Data Availability

Upon a reasonable request, the corresponding author will provide data supporting the findings of this study.
